# miRdentify: high stringency miRNA predictor identifies several novel animal miRNAs

**DOI:** 10.1093/nar/gku598

**Published:** 2014-07-22

**Authors:** Thomas B. Hansen, Morten T. Venø, Jørgen Kjems, Christian K. Damgaard

**Affiliations:** 1 Department of Molecular Biology and Genetics (MBG), Aarhus University, Aarhus, Denmark; 2 Interdisciplinary Nanoscience Center (iNANO), Aarhus University, Aarhus, Denmark

## Abstract

During recent years, miRNAs have been shown to play important roles in the regulation of gene expression. Accordingly, much effort has been put into the discovery of novel uncharacterized miRNAs in various organisms. miRNAs are structurally defined by a hairpin-loop structure recognized by the two-step processing apparatus, Drosha and Dicer, necessary for the production of mature ∼22-nucleotide miRNA guide strands. With the emergence of high-throughput sequencing applications, tools have been developed to identify miRNAs and profile their expression based on sequencing reads. However, as the read depth increases, false-positive predictions increase using established algorithms, underscoring the need for more stringent approaches. Here we describe a transparent pipeline for confident miRNA identification in animals, termed miRdentify. We show that miRdentify confidently discloses more than 400 novel miRNAs in humans, including the first male-specific miRNA, which we successfully validate. Moreover, novel miRNAs are predicted in the mouse, the fruit fly and nematodes, suggesting that the pipeline applies to all animals. The entire software package is available at www.ncrnalab.dk/mirdentify.

## INTRODUCTION

MicroRNAs (miRNAs) are small evolutionary conserved non-coding RNAs involved in the regulation of gene expression by translational repression and mRNA destabilization (1,2). This regulatory role has within the last decade been shown to be crucial for most biological processes including developmental timing, stem cell differentiation, cell division and disease development (3–6). The biogenesis of miRNAs includes two cleavage events carried out by RNase III enzymes, Drosha and Dicer, producing an RNA duplex structure with 2 nt 3′ overhangs at each terminus. Typically, one strand—the guide strand—from the RNA duplex is incorporated into the RNA-induced silencing complex, which represents the miRNA effector complex, whereas the other strand—the passenger strand—is degraded. Using highly sensitive deep-sequencing techniques, miRNA biogenesis intermediates (i.e. passenger strands) are in most cases easily detectable; thus, for each bona fide miRNA, both duplex-forming guide and passenger strand reads with the corresponding RNase III-specific overhangs should be detected in contemporary datasets.

Within the last decade, great efforts have been made to predict and identify novel miRNAs within the genomes of eukaryotes. A handful of tools have been developed to predict miRNAs in genomes either using a comparative phylogenetic approach (7,8) or a non-comparative, support vector machine-based approach (9–11). With the emergence of high-throughput sequencing techniques, a great resource of small RNA species within cells is readily available, which greatly enhances its predictive power. This has been implemented in prediction algorithms to identify novel miRNA species more efficiently (12–16). Currently, miRDeep2, which scores the likelihood of novel candidates according to Bayesian probabilities, is likely the most reliable prediction tool available (13,17).

Measures to ensure confident animal miRNA annotation (18) have essentially been based on two main criteria: (i) detectable expression and (ii) proper structural features, i.e. a stem-loop structure conforming to Drosha/Dicer-dependent maturation. However, with the depth of high-throughput sequencing, the criterion of expression is easily met, and without increased stringent structural specificity, a multitude of false-positive miRNA species will inevitably emerge on the global scale. Hence, exact parameters for miRNA hairpin recognition have never been established and with the current depth of small RNA sequencing techniques and the abundant occurrence of putative stem-loop structures in animal genomes (19), the confidence of miRNAs annotated solely based on sequencing efforts is in several cases highly questionable (20). Accordingly, several studies have identified ‘non-genuine’ miRNA entries in the miRBase registry, which cannot be validated experimentally (21), or seem to be derived from degradation of abundant RNA species (22,23). This emphasizes the imminent need to improve the reliability of miRNA annotation and thus additional parameters and increased stringency are necessary to reduce continued annotation of non-genuine miRNAs.

Here, we have developed a stringent approach to confidently predict novel miRNAs in animals. Only miRNA hairpin structures with distinct guide and passenger strand reads, annotated miRNAs as well as predicted, putatively engaging in duplex formation are considered in the analysis. In addition, we establish a series of distinct criteria serving as dynamic parameters for novel miRNA prediction. The strength of all parameters is concomitantly increased until the estimated false-positive rate (FPR) is below 0.01. The outcome of this approach is a high confidence and stringent miRNA prediction tool, which we coin miRdentify. As a result of accumulating multiple available small RNA sequencing datasets, we predict more than 400 novel human miRNAs including the first human male-specific miRNA, which we validate by northern blotting. Furthermore, miRdentify also predicts several novel miRNAs in the mouse, the fruit fly and nematodes. miRdentify is publicly available at www.ncrnalab.dk/mirdentify.

## MATERIALS AND METHODS

### miRdentify

Small RNA sequencing datasets were retrieved from the Gene Expression Omnibus (GEO); accession numbers are listed in Supplementary Table S1. The reads were adaptor-trimmed and collapsed using miRdentify (the fa2tab tool) discarding all reads not within 18–26 nucleotides in length. Reads were subsequently mapped to reference genome (GRCh37/hg19, GRCm38/mm10, BDGP R5/dm3, WS220/ce10) using the Bowtie version 1.0.0 (24).

**Table 1.** The number of novel miRNAs predicted in human, mouse, fruit fly and nematodes, and the total small RNA sequencing reads used in the prediction

Organism	Ref. Genome	Total datasets	Total reads (×10^6^)	Novel miRNAs
***Homo sapiens*** (dataset 1)	GRCh37/hg19	355	1998	420
***Homo sapiens*** (dataset 2)	GRCh37/hg19	50	747	94
***Mus musculus***	GRCm38/mm10	149	791	109
***Drosophila melanogaster***	BDGP R5/dm3	87	750	23
***Caenorhabditis elegans***	WS220/ce10	45	110	46

Detailed information is available in Supplementary Tables S1–S3.

Mapped reads within 46–80 nucleotides (the predefined size of pre-miRNA hairpin, Supplementary Figure S1A) were evaluated in duplex formation using MultiRNAFold version 1.1 (25). A locus containing reads capable of duplex formation with minimal free energy (MFE) ≤ −14, consistent with the vast majority of annotated 5p and 3p duplexes (Supplementary Figure S1B), were kept for further analysis as miRNA candidates. The most abundant read in the candidate loci was assigned the guide strand, and the most abundant read able to potentially produce 46–80 nt hairpin structures with the guide strand was assigned passenger strand. The pre-miRNA locus was then defined by the guide and passenger strand adding 10 additional nucleotides before and after (useful for more accurate structural prediction and overhang assessments). All pre-miRNA loci were scored in 10 parameters (Figure 2 and Supplementary Figures S2–S7) and divided into annotated miRNA based on miRBase version 20 (26) or candidate miRNA. For each parameter, a cutoff was assigned based on the performance of annotated miRNAs, e.g. excluding one percent of annotated miRNAs. The strength of the parameters was determined by the number of candidate miRNAs excluded by the given cutoff (see text for more details). Subsequently, the number of expected positives and the associated FPR was calculated, as follows (see also Supplementary Figure S8):

\begin{equation*} FPR = \frac{{{\rm Expected}}}{{{\rm Observed}}} = \frac{{\Pi (1 - {\rm Strength})xN}}{{{\rm Observed}}},\end{equation*}

where Observed is the number of candidates passing all cutoffs and *N* is the total number of candidates considered. Cutoffs for each parameter were then incremented uniformly (i.e. excluding 2, 3, 4%… of annotated miRNAs) until an FPR ≤0.01 was reached. In the end, a final list of novel miRNAs is compiled comprising all the candidate miRNAs successfully passing all 10 parameter cutoffs.

**Figure 1.**
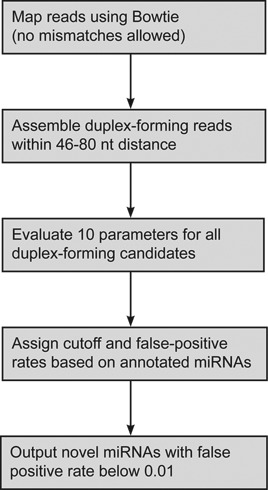
The miRdentify pipeline.

**Figure 2.**
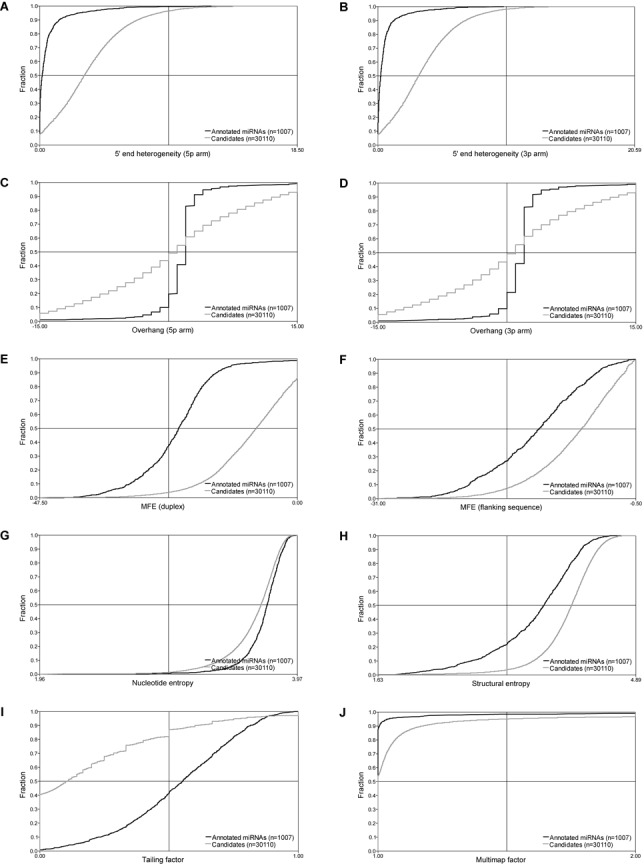
Parameters. (**A**–**J**) To distinguish true miRNA entities from other hairpin-forming structures, annotated miRNAs (black, *n* = 1007) and all the putative candidates (gray, *n* = 30 110) were scored by 5′ heterogeneity (A, B), overhangs, negative numbers indicate 5′ overhang (C, D), thermodynamics (E, F), entropy (G, H), tailing (I) and multimapping (J). Scores are depicted as cumulative plots. In all cases, miRNA and candidates differ significantly (*P* < 2.2E-16, two-sided Kolmogorov–Smirnov test). See also supplementary Figures S2–S7 for detailed information on parameter value assessments.

### Quality assessment of other methods

Shell commands used are listed in the Supplementary Table S4. Rfam percentage was assessed by mapping the mature miRNA sequences against Rfam version 11. Repeatmask percentage was calculated based on the mask from UCSC (University of California Santa Cruz) genome browser. Here, miRNAs were scored as repeatmasked, if more than half the pre-miRNA locus was embedded in a repeatmasked region. Pre-miRNA sequence including 10 nucleotides flanking sequence on each side of the pre-miRNA subsequently submitted to available low-throughput miRNA prediction tools: miR-Abela (http://www.mirz.unibas.ch/cgi/pred_miRNA_genes.cgi), miREval 2.0 (http://mimirna.centenary.org.au/mireval/) and CID-miRNA (http://mirna.jnu.ac.in/cidmirna/) scores were retrieved from their respective online webtools, using default setting. miRFinder version 4.0 (http://www.bioinformatics.org/mirfinder/), miRPara 6.2 (http://159.226.126.177/mirpara/download.htm) and MiPred (http://www.bioinf.seu.edu.cn/miRNA/) were downloaded and executed. Finally, the output produced by the different algorithms was retrieved and analyzed.

### miRNA validation

The novel two chrY-situated miRNAs, NM#419 and NM#420, were PCR amplified with ∼200 bp flanking sequence on each side using ATAGAAGCGGCCGCGCCTCTTTCTAGCCATTGGA/ATAGAAGTCGACAGAATTGTCTTGGGCCACAC or ATAGAAGCGGCCGCTTTGTGAGGCAGCAAAGACA/ATAGAAGTCGACTGTTGGGTTGATAGGTGCAG, respectively, digested with NotI and SalI and inserted into NotI/SalI-digested pJEBB vector. HEK293 cells were transfected with either empty pJEBB or miRNA containing pJEBB using calcium phosphate procedure. Forty eight hours after transfection, RNA was harvested using TRIzol^®^ reagent (Invitrogen) according to manufacturer's protocol. Thirty micrograms of RNA was separated on 12% denaturing polyacrylamide gel electrophoresis and transferred to Amersham hybond^TM^-N+ membranes (GE Healthcare). The membranes were then hybridized in Church buffer [0.5 M NaPO_4_, 7% sodium dodecyl sulphate (SDS), 1 mM ethylenediaminetetraacetic acid (EDTA), 1% bovine serum albumin (BSA), pH 7.5] with ^32^P-endlabeled DNA probes (NM#419, GGTAACTTCCCAACATAGTATT; NM#420, CTCACAAACTGCTTCAAAAGCA; miR-15b, TGTAAACCATGATGTGCTGCTA) at 37°C. Membranes were washed three times in 2xSSC (300 mM NaCl, 30 mM Na-citrate, pH 7.0) with 0.1% SDS at room temperature and exposed on phosphorimager screens and analyzed using Quantity One^®^ or Image Lab^TM^ software (Bio Rad).

## RESULTS

Previously, it has been shown that the majority of bona fide miRNAs are detectable across multiple deep sequencing datasets (20). Furthermore, consolidation of datasets enriches for distinct biological entities and dilutes random degradation products. In an attempt to identify novel miRNA using online datasets, we collected and combined ∼350 small RNA sequencing datasets, resulting in ∼3 billion total reads in the size range between 18 and 26 nucleotides (Supplementary Table S1).

### Parameters for *in silico* miRNA annotation

An imperative task for confident *in silico* annotation of miRNAs is stringency. While potentially sacrificing several bona fide miRNAs, we aim to avoid false positives using a highly stringent approach with several ‘cutoff criteria’ that should all be met in order to become scored as a miRNA. First, all reads from the consolidated datasets were mapped to the human genome allowing no mismatches (hg19), and discarding reads with more than four genomic hits to avoid highly repetitive loci (Figure 1). This resulted in mapping of ∼82% of all reads (73% mapped onto known human miRNA loci, miRBase version 20).

The detection of both 5p and 3p arms (miR and miR* sequences) in miRNA prediction has previously been reported as an important signature indicative of miRNA-like biogenesis (27,28). Thus, reads with potential duplex formation capabilities were collected by ‘walking’ along the genome in 46–80 basepair windows, including all the additional reads emanating from the same locus (Figure 1). This resulted in 31 117 putative candidates. Cross-referencing the list of candidates with miRBase version 20 revealed that 1007 were annotated miRNAs and the remaining 30 110 candidates were, thus, considered as putative novel miRNAs. For each candidate, 10 different features were analyzed (Figure 2A–I).

#### Heterogeneity

In order to avoid degradation-derived reads positioned on miRNA-like hairpin structures to be included in the final list of confident miRNAs, we established a simple measure of heterogeneity. Firstly, miRNA biogenesis and downstream events generally result in a high precision of the miRNA 5′ end in agreement with seed-based miRNA-targeting. Therefore, low 5′ heterogeneity is typically observed for bona fide miRNAs, in agreement with RNase III-based endonucleolytic cropping (27,29). Heterogeneity was calculated for each arm as the count of each read mapping the respective arm multiplied by the distance to the most predominant read relative to total count (Supplementary Figure S2). As expected, heterogeneity was radically lower for loci matching annotated miRNAs compared with the list of putative miRNA candidates retrieved in the initial genome survey (Figure 2A and B). Furthermore, annotated miRNAs exhibit almost identical 5p and 3p arm heterogeneity. The 5′ end of the 3p arm is supposedly both Drosha and Dicer dependent, and therefore expected to be more prone to heterogeneity. However, the lack of increased heterogeneity in the 3p arm suggests that either Dicer cleavage is not solely defined by a molecular ruler as previously suggested (30,31) or that Drosha/Dicer both conduct accurate processing but post-processing events are responsible for the observed heterogeneity.

#### 3′ overhang

Bona fide miRNA duplex structures are typically characterized by two nucleotide 3′ overhangs at both ends due to the processing mechanisms of Drosha and Dicer. As noted above, the occurrence of predominant reads corresponding to either the 5p or 3p arm of the miRNA hairpin is included here as a prerequisite for *in silico* miRNA prediction, which enables an overhang assessment of the predicted 5p/3p duplex formation. We evaluated the overhang profile of annotated miRNAs (Supplementary Figure S3) and as expected we identified the two nucleotide overhang as the most abundant type of overhang (Figure 2C and D). In contrast, the list of candidates showed a wide range of overhangs with no obvious preference, which likely represent non-RNAi-derived products. This indicates that overhang discrimination is a useful parameter to distinguish between bona fide miRNAs and random hairpin structures.

#### Thermodynamics

miRNA hairpin structures exhibit a lower free energy than random RNA (32), and therefore we included a thermodynamic MFE score as a parameter (Supplementary Figure S4). Here, we not only determined the calculated MFE of miRNA duplex structures (Figure 2E, Supplementary Figure S4A and B) but also the MFE of the flanking region (Figure 2F, Supplementary Figure S4A and C), which has been shown to be part of the recognition by the microprocessor (33). As expected, annotated miRNAs display a lower MFE in the duplex structure and flanking region compared to the group of putative candidates.

#### Entropy

Hairpin structures of simple repeat RNA are widespread in the transcriptome and therefore often found in small RNA sequencing datasets. Even though miRNA-like biogenesis and function of simple-repeat RNA has been observed, we included a nucleotide complexity assessment to avoid potential erroneous miRNA prediction of simple repeat hairpins (Supplementary Figure S5A and B). Indeed, annotated miRNAs exhibit a higher degree of nucleotide entropy compared to the list of putative candidates (Figure 2G), suggesting that bona fide miRNAs tend to have a more complex nucleotide arrangement than the average candidate hairpin structure. Also, structural entropy was similarly assessed (Supplementary Figure S5A and C). Conventionally, the pre-miRNAs are simple hairpin-loop structures, and, as seen in Figure 2H, the structural entropy of annotated pre-miRNA hairpins is generally lower than candidate hairpins, suggesting a preference for lower structural complexity in the secondary structure of miRNA precursors.

#### Non-templated tailing

Several studies have shown non-templated tailing of miRNAs with adenosine or uridine residues (34–36). Consistently, these 3′end modifications are widespread in small RNA sequencing datasets and we also observe a relatively high level of tailing in the group of annotated miRNAs compared to the group of candidates (Figure 2I) as measured by the ratio of genomic non-mapped reads having a terminal A or U mismatch compared to reads with mismatches elsewhere (Supplementary Figure S6).

#### Multimapping

Finally, we excluded all provisional candidates containing ‘multimapped’ reads to avoid ambiguous miRNA evidence. In the initial perfect mapping of reads, a restriction of no more than four genomic hits was set to discard highly repetitive loci. Here, the discarded reads (i.e. reads with more than four genomic hits) are mapped back onto the candidate sequences, and the fraction of multimapping reads relative to single-mapping reads (i.e. reads with no more than 4 genomic hits) determines the multimapping score (Supplementary Figure S7). This reveals the candidates that have undergone an incomplete evaluation due to the initial multimap restriction and therefore the assignment of guide and passenger strand and the downstream analysis of the high-scoring candidates are therefore not trustworthy. Also, annotated miRNAs rarely emanate from loci with a high multimap occurrence (Figure 2J), suggesting that this parameter not only addresses the trustworthiness of the above assessments but also distinguishes between true and false miRNA loci.

### Cutoff values and FPRs

**Figure 3.**
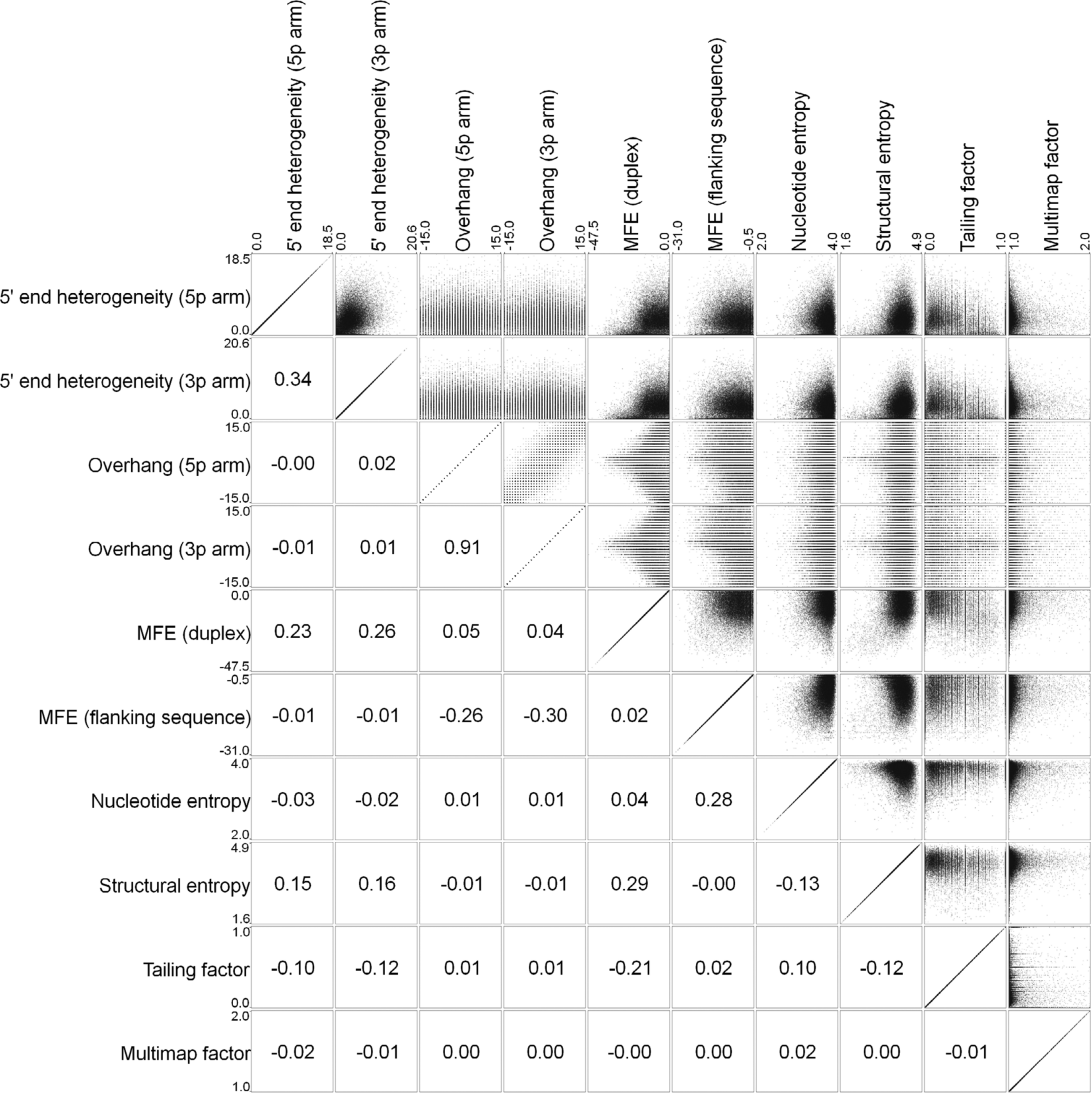
Parameter dependencies. Scatter plots and Pearson correlation scores for all parameter pairs. Annotated miRNAs are excluded (*n* = 30 110).

To estimate the number of miRNAs predicted by chance and thereby calculating the FPR, each parameter has to behave independently. To validate this assessment, Pearson correlations between all parameters were calculated (Figure 3). This showed, not surprisingly, that the 5p and 3p overhang values were highly interdependent. To a lesser degree, a positive correlation between 5p and 3p heterogeneity and between the structural entropy and duplex MFE is observed. Also, the thermodynamic properties of the flanking sequence and the entropy of nucleotide composition show a positive correlation. However, in this case, the correlation is complementary, as a low flanking MFE and high nucleotide entropy are known features of bona fide miRNAs. This means that the strength of flanking MFE and nucleotide entropy combined, exceeds the product of individual strengths, i.e. these parameters in conjunction act synergistically in demarcating miRNA species from non-miRNA hairpin candidates. Conclusively, we approximate that all parameters except the overhangs behave independently, and thus for the FPR assessment, the overhangs are grouped together, resulting in nine independent parameters.

**Figure 4.**
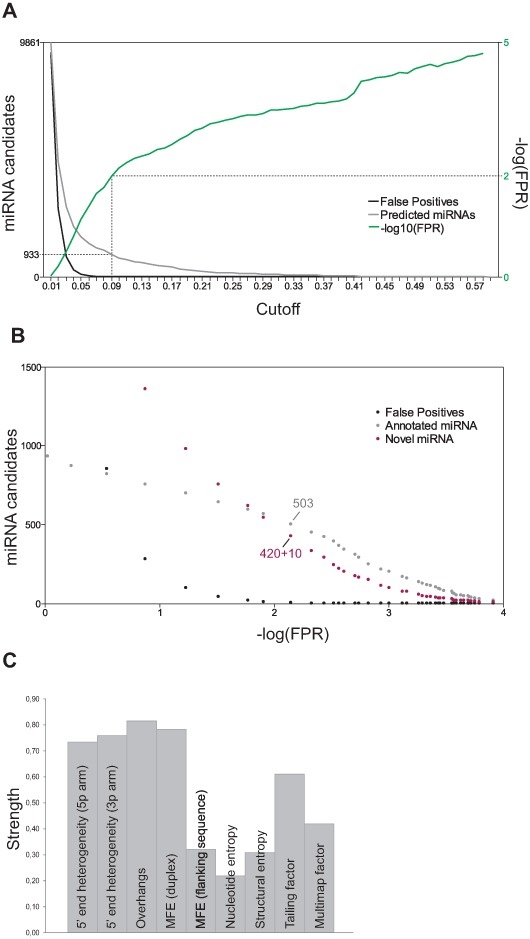
False positive rate. (**A**) Using variable relative cutoffs (*X*-axis), the expected false positives (black) and the observed number of predicted miRNA (gray) are plotted. The –log10 ratio between false positive and the actual number of predictions is plotted in green. By default, a –log10 (prediction miRNA/expected false positives) above 2 (i.e. an FPR ≤0.01) was used corresponding to a relative cutoff of 0.09. (**B**) Scatterplot with annotated (gray), observed positive candidates (red) and expected false positives (black) as a function of FPR. 430 novel miRNAs of which 10 are antisense to known miRNAs loci and 503 annotated miRNAs pass the FPR ≤0.01 criterion. (**C**) Using the 0.09 cutoff, the strength of each parameter is depicted as the fraction of candidates failing the specific parameter cutoff. The cutoff values and the corresponding strength for each parameter are depicted in Supplementary Figure S8A and B.

Then, starting at 1% relative cutoff, for each parameter the worst 1% of annotated miRNAs was discarded, i.e. the miRNAs with high heterogeneity, adverse overhangs, low level of tailing, low nucleotide entropy etc. The absolute values in each parameter corresponding to a 1% cutoff were used to define true and false miRNAs (Supplementary Figure S8A, first column). As an example, 1% of annotated miRNAs detected has a 5′end heterogeneity score above 6.48 (Supplementary Figure S8A), whereas 13% of all the collective miRNA candidates exceed this score. Therefore, at 1% cutoff, the strength of this parameter is 0.13 (number of eliminated candidates/total number of candidates, Supplementary Figure S8B). Applying this approach on the other parameters, the expected and observed number of candidates passing all parameters were 9423 and 9861, respectively (Supplementary Figure S8B and C), resulting in an FPR of 0.956 (expected/observed, Supplementary Figure S8B and C). Subsequently, the relative cutoff was incremented stepwise by 1% (Figure 4A and Supplementary Figure S8A and B). For each cutoff, the ratio between expected (Figure 4A, black line) and observed miRNA candidates (Figure 4A, gray line) denotes the FPR (Figure 4A, green line). We selected an FPR ≤0.01 to confidently demarcate false and true positives. This was obtained at a relative cutoff of 9% (0.09, resulting in an FPR of 0.007, Figure 4A and Supplementary Figure S8B) producing a total of 933 accepted miRNA structures (Figure 4A), of which 503 species were annotated in miRBase, 10 species were antisense to annotated miRNAs and the remaining 420 were currently not annotated and thus considered to be novel miRNAs (Figure 4B). As expected, based on the comparison between annotated and candidate miRNAs (Figure 2), 5′heterogeneity, duplex thermodynamics, overhangs and tailing scored as the strongest parameters in the miRNA prediction (Figure 4C), discarding between 60 and 80% of candidate structures at the 9% cutoff.

### Comparison to other prediction methods

**Figure 5.**
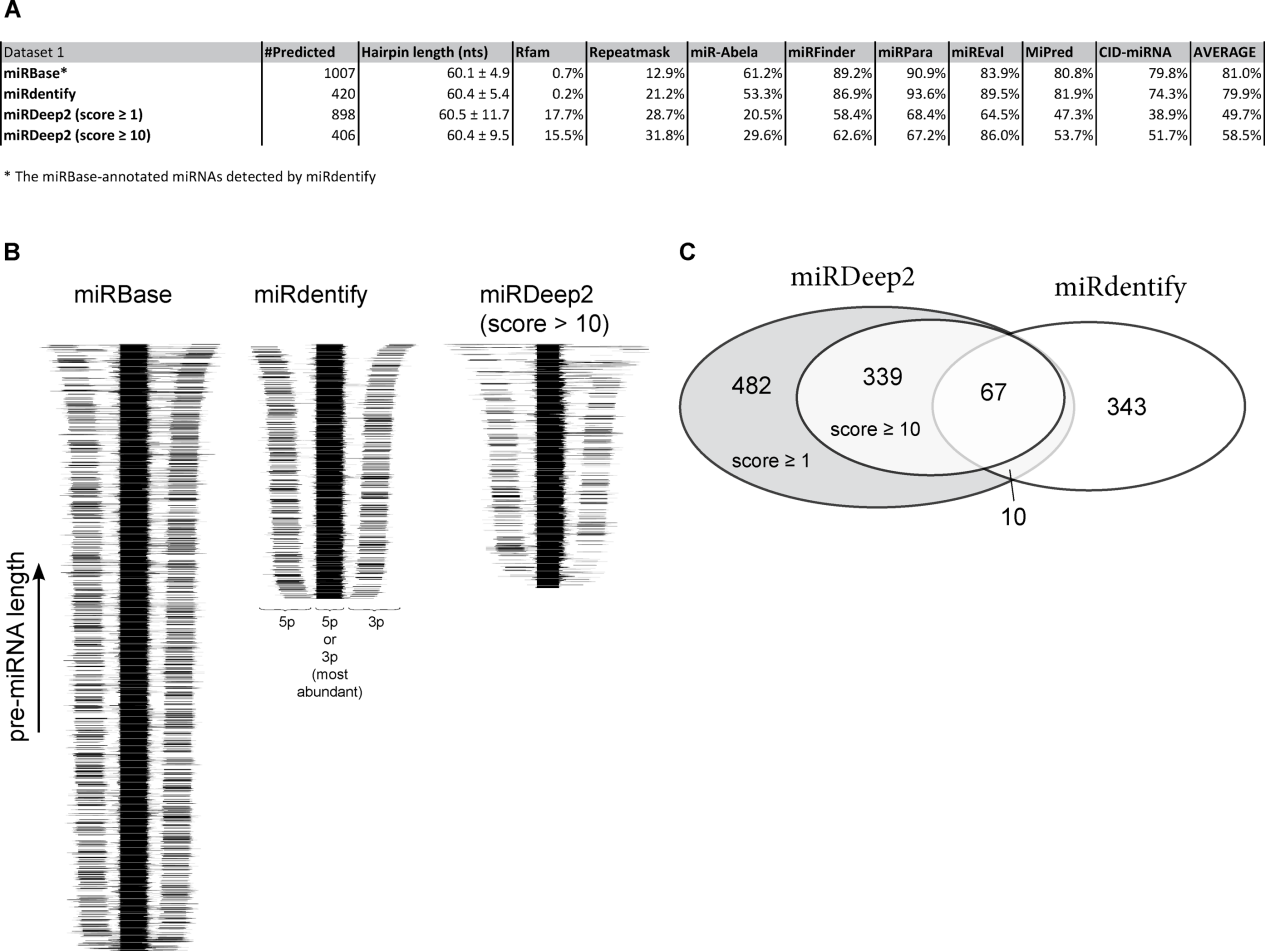
Comparison to miRDeep2. (**A**) Table listing various features of annotated miRNAs, miRDeep2-predicted miRNA and miRdentify-predicted miRNAs. (**B**) Distribution of reads on predicted pre-miRNA. Reads are mapped perfectly (no mismatch allowed) onto the predicted species and aligned to the 5′ end of the most abundant read. For each pre-miRNA locus, read density is shown relative to the most abundant read and depicted in gray-scaled intensities. (**C**) Venn diagram depicting overlapping prediction between miRDeep2 and miRdentify.

Currently, miRDeep2 is one of the most reliable methods for prediction of novel miRNAs. Based on the consolidated small RNA dataset, miRDeep2 was able to predict between 898 and 406 novel miRNAs using a miRDeep2 score ≥1 or ≥10, respectively (Supplementary Table S2). However, the set of novel miRNAs predicted by miRDeep2 was more heterogeneous in terms of pre-miRNA length compared to miRdentify and miRBase-annotated miRNAs (Figure 5A and Supplementary Figure S12D), and contained a considerably higher percentage of Rfam database-associated RNA species (Figure 5A). Furthermore, a higher fraction of miRNAs predicted by miRdentify was scored as bona fide when subjected to six previously established low-throughput prediction tools available (Figure 5A): miR-Abela (10), miRFinder (37), miRPara (38), miREval (39), MiPred (40) and CID-miRNA (41). Surprisingly, based on these analyses, little or no difference between the high scoring (score ≥10) and total (score ≥1) miRDeep2 species was observed, suggesting that increased score does not infer increased quality. Comparing the distribution and density of reads on the predicted pre-miRNA loci showed a very stringent pattern of 5p and 3p reads on the pre-miRNAs predicted by miRdentify similar to annotated miRNA (Figure 5B), whereas the miRDeep2 predicted species displayed a more heterogeneous distribution, and accordingly only a small overlap between the two prediction tools was observed (Figure 5C). Conclusively, even though the stringent prediction employed by miRdentify results in a reduced number of predicted miRNAs, the comparison indicates that the quality and true-positive rate may be significantly higher than miRDeep2 irrespective of the miRDeep2-score cutoff used.

To further evaluate miRdentify, we also compared our pipeline with the following prediction methods: miRDeep_star (16), miRanalyzer (14) and miReap (http://sourceforge.net/projects/mireap). Unfortunately, using our hardware, none of these methods were able to complete a prediction on the complete dataset 1. Therefore, to enable a meaningful comparison, we used a sub-dataset, dataset 2 (Table 1 and Supplementary Table S1) comprising 50 sRNA sequencing datasets giving rise to a total read count of ∼750 million reads. In the prediction by miRDeep2 and miRDeep_star, we only considered candidates with score above 10. In case of miRanalyzer, the ‘Unlikely/No dicer’ group of candidates was excluded, and with miReap only candidates with 5p and 3p reads were considered. The comparison showed, as with dataset 1, that miRdentify was superior in terms of quality, but modest in quantity (Supplementary Figure S10A and Supplementary Table S2). Furthermore, read density was more distinct with miRdentify (Supplementary Figure S10B). Again, the overlap between predictions was surprisingly small; only 16 species predicted by all five methods (Supplementary Figure S10C). However, miRdentify exhibits the lowest percentage of uniquely predicted miRNAs, i.e. the number of miRNA only predicted by one method, suggesting that most miRNAs predicted by miRdentify are reproducibly predicted by at least one other method. Finally, we compared the duration of the prediction (Supplementary Figure S10D). Here, miRdentify and miReap were by far the fastest methods completing a prediction on ∼750 million reads in less than 1 h.

We next compared the fraction of annotated miRNAs having an FPR above or below 0.01, respectively (Supplementary Figure S11A). This clearly showed a much larger fraction of approved miRNAs in the below 0.01 fraction. Furthermore, as noted above, the 5′ heterogeneity parameters exhibited a somewhat positive correlation suggesting that they do not behave completely as independent parameters. Thus, we performed a prediction with merged 5′ heterogeneities (Supplementary Figure S11B). As expected, this revealed a reduced number of predicted miRNAs, but with similar scores as the original prediction, suggesting that assuming independent 5′ end heterogeneity parameters does not interfere with the general quality of the prediction.

### Validation of chrY-specific miRNAs

**Figure 6.**
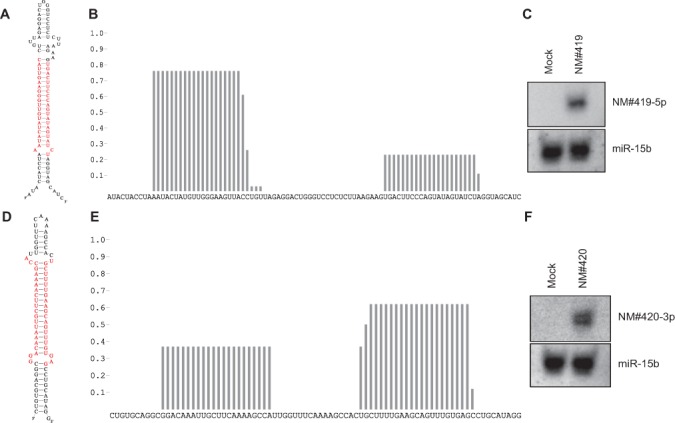
Validation of chrY-specific miRNAs. (**A** and **D**) Predicted secondary structure of NM#419 (hg19: chrY:16811019–16811092,+) (A) and NM#420 (hg19: chrY:4020820–4020881,-) (D) with 5p and 3p arms marked in red. (**B** and **E**) Distribution of reads on the NM#419 locus (B) and on the NM#420 locus (E). (**C** and **F**) Northern blot with RNA from HEK293 cells transfected with either empty vector (EV) or a vector expression, the candidate miRNA. Northern membrane was probed against the predominant NM#419-5p arm (C) or the NM#420-3p arm (F), and miR-15b as a loading control.

Two novel miRNA genes (NM) positioned on chrY were predicted (NM#419 and NM#420, Figure 6). To our knowledge, all annotated chrY-encoded miRNAs in humans also have a chrX-encoded counterpart, and therefore no chrY/male-specific miRNA has ever been identified. NM#419 is exclusively positioned on chrY with no apparent sequence homology elsewhere in the genome, whereas NM#420 has a near-perfect region of homology on chrX, allowing the mature sequence to also originate from chrX. Nonetheless, to validate this prediction, the two miRNAs were cloned as pri-miRNAs into a mammalian expression vector and transfected into HEK293 cells. After 48 h, the RNA was analyzed by northern blotting and probed for the mature miRNA strand. Here, both pri-miRNAs produced ∼22 nt RNA species efficiently (Figure 6C and F), suggesting that they are indeed genuine miRNAs. Therefore, to our knowledge this is the first identification of a male-specific miRNA in humans. Determining the expression profile and biological relevance of the chrY miRNA awaits further analyses.

### Predicting long miRNAs

In the above prediction, only pre-miRNA hairpins in the range of 46–80 nucleotides were considered, which also includes the vast majority (97%) of annotated miRNAs in miRBase (Supplementary Figure S1A). However, to our knowledge, there is no size restriction in the biogenesis of miRNA *per se*, and therefore it could easily be envisioned that a class of larger pre-miRNA (i.e. 81–120 nucleotides) structures exist. As no parameters in the above analysis have any inherent size preference, we speculated that miRdentify would also confidently predict long pre-miRNA. In this case, since only a few annotated pre-miRNAs in the 81–120 size range exist, an alternative approach must be established to determine the cutoff values. Hence, we instead fixed the relative strengths of each parameter obtained in the previous prediction and then as before increased the stringency gradually. As a result, based on the similar criterion for demarcation, i.e. FPR ≤0.01, we only found 12 candidates (Supplementary Figure S12A), in which two candidates are already annotated in the miRBase (miR-3678 and miR-5010) and two other candidates are consistent with tailed mirtron biogenesis as judged by the location of annotated splice sites (data not shown, Supplementary Figure S12B). Also, nine of the 12 candidates were below 92 nucleotides in length indicating a preference for shorter species (Supplementary Figure S12B). Furthermore, conducting a similar prediction on 121–160 nucleotide species, no novel miRNAs passed in almost 10 000 initial candidates. Alternatively, addressing pre-miRNAs in a full 46–160 size range, miRdentify predicts 436 novel miRNAs, of which 406 overlap with the 46–80 nucleotide prediction (Supplementary Figure S12C). Finally, comparing the length distribution of 46–160 nucleotide predicted pre-miRNAs with miRBase entries having 5p and 3p annotation, no significant difference is observed, whereas the miRDeep2 prediction differs significantly (Supplementary Figure S12D). Conclusively, this argues that pre-miRNAs in general are between 46 and 80 nucleotides in length, and additionally, strengthens the confidence of miRdentify predictions.

### Applying miRdentify to other animals

Performing miRNA prediction in other animals, we successfully identified 109 novel miRNAs in the mouse (*Mus musculus*), 20 in the fruit fly (*Drosophila melanogaster)* and 46 in nematodes (*Caenorhabditis elegans)* (Table 1, Supplementary Table S3). Across the species, it is evident that the parameter strengths are comparable (Supplementary Figure S13A and B) in agreement with the notion that animal miRNAs are roughly similar in structure (7). However, the tailing parameter constitutes one noteworthy difference between organisms. Based on our analyses, tailing seems to be much less apparent in the fruit fly and nematodes (Supplementary Figure 13A). This either reflects low background tailing, i.e. the average tailing observed on the candidate mapped reads, in which case tailing efficiently demarcates true and false miRNAs as seen in the fruit fly and therefore has a high strength (Supplementary Figure S13B), or it reflects low miRNA-specific tailing as seen in nematodes (Supplementary Figure S13B). In fact, most nematode miRNAs seem to avoid tailing compared to the bulk of candidate structures (Supplementary Figure S13C), which was somewhat surprising as a TUT4 ortholog, Pup-2, was recently identified and characterized to uridylate pre-let-7 in nematodes (42). However, in this particular case, tailing seems to be LIN28-pre-let-7-specific and inhibits Dicer processing. Also, background tailing was observed to be much more frequent in *C. elegans* (0.44) compared to the other organisms (0.22, 0.24 and 0.11 in human, mouse and fruit fly, respectively, Supplementary Tables S2 and S3, tailing average), suggesting that tailing here fails as a strong parameter to select for genuine miRNAs.

Moreover, the requirement for an extended flanking stem structure seems also to be more pronounced in mouse and human pri-miRNAs (Supplementary Figure S13B). Perhaps with increasing genome complexity, the affinity of the microprocessor toward pri-miRNAs hairpin structures has increased in stringency to avoid adverse processing of miRNA-like hairpins.

## DISCUSSION

Here, we have established a transparent and simple miRNA prediction strategy coined miRdentify that, based on gradual increase in 10 parameters, ensures a selection of highly miRNA-like hairpin structures. In fact, the stringency of this strategy discards 50% of the annotated miRNAs with 5p and 3p detectable reads and therefore serves as a very conservative approach crucial for bona fide novel miRNA prediction based on contemporary high-throughput small RNA sequencing datasets.

Expectedly, the necessary cost of a high stringency approach is sensitivity, i.e. the rate of false negatives. Increasing the stringency of novel miRNA detection in response to the large sequencing depth of current sequencing technologies is crucial, as any acceptance of false positives has the potential to undermine the credibility of the miRNA field and the miRBase. Thus, we argue that miRNA annotation based solely on sequencing must be performed conservatively. Attempting to predict all miRNAs expressed in any given sequencing dataset would almost certainly include high quantities of false positives and the quality of the prediction would consequently be lowered. Adhering to a conservative prediction, non-canonical miRNAs such as miR-451 (43,44) would be disregarded. We suggest that any unconventional miRNAs must instead be predicted using other algorithms and subsequently validated experimentally.

Even with the high level of stringency attained with miRdentify, we report hundreds of novel miRNAs, 420 in humans, 109 in mouse, 23 in fruit fly and 46 in nematode. We observe that the parameters used for miRNA prediction behave in a comparable manner between animal species except for tailing and to some degree the structure of the flanking region. In situations where a specific parameter does not effectively select for bona fide miRNAs, it may be beneficial to exclude the parameter in the given prediction; however, an ineffective demarcation between miRNAs and other hairpin species also means low parameter strength and thus only a minor contribution to the overall FPR assessment and downstream novel miRNA output. Therefore, we argue that the dynamic strengths of parameters in miRdentify comprise a flexible tool for miRNA prediction in various organisms. Moreover, implementation of additional parameters useful for miRNA prediction is easily achieved in the miRdentify pipeline, which makes this a compliant tool that enables adaptation of future miRNA biology insights.

Interestingly, miRdentify predicts two chrY-encoded miRNA, which we successfully validate experimentally, and this constitutes the first reported truly male-specific miRNA. Although they exhibit no evolutionary conservation, which makes *in silico* target prediction much more difficult, future studies will decipher the functional role and relevance for these miRNAs.

The prediction by miRdentify, compared with other methods, is generally higher in quality but lower in numbers. Concerning the sensitivity and recall rate of annotated miRNAs, miRdentify identifies 1007 annotated species, of these 503 species have FPR below 0.01, indicating a sensitivity of ∼50%. Therefore, as expected for a conservative and stringent approach, it does not exhaust the repertoire of undiscovered miRNAs in the genome. In comparison, miRDeep2 recovers 1010 annotated miRNAs, of which 689 have a score below 10, thus a ∼68% sensitivity. In dataset 2, miRdentify detects 823 and recalls 340 (41%) annotated miRNAs with an FPR below 0.01; miRDeep2 and miRDeep_star detect 1019 and 958, and recall 386 (38%) and 398 (42%), respectively, with scores above 10 (Supplementary Table S2). At least for dataset 2, this shows a very similar level of sensitivity once the miRNA has been detected. However, miRdentify detects fewer annotated species in total, which primarily is due to the 5p/3p duplex requirements, where in contrast several annotated miRNAs found by miRDeep2 and miRDeep_star only have reads on one arm. Moreover, perfectly mapped reads (i.e. no mismatch tolerance) are necessary for initial detection in miRdentify. Here, miRDeep2 also identifies miRNA species, novel as well as annotated, without the presence of any perfectly mapped reads in the dataset, and this is also the case for miRanalyzer, which, at least for ∼22 nt RNA species, makes the accuracy of the prediction more dubious. Thus, the high stringency of miRdentify impacted only modestly the sensitivity compared to miRDeep2 and miRDeep_star, suggesting that the reduced quantity of predicted miRNAs reflects more accurately the abundance of still unannotated true miRNAs.

MiRDeep2 and miRDeep_star attribute each putative miRNA with a score. Here, we used a score threshold of 10 to select for the highest quality candidates, even though a score of 1 or 0 has been recommended by the authors (12,16). Using score 0 as threshold, 1182 and 1525 novel miRNAs are predicted from dataset 2 by miRDeep2 and miRDeep_star, respectively, and in the latter case, a large subset of candidates between score 0 and score 10 only have reads on one arm of the pre-miRNA hairpin (data not shown, Supplementary Table S2). MiRanalyzer and miReap are not scoring the candidates and instead read count is the only available feature by which candidates are sorted; however, miRanalyzer groups the prediction into four categories dependent on read density signature, whereas miReap outputs all candidates in one list. MiRdentify assigns each candidate with an FPR estimate, and as noted above, we suggest an FPR below 0.01 to define novel miRNAs (all outputs are available in Supplemental Table S2). Furthermore, miRdentify outputs all the parameter values for each miRNA candidate and therefore it is clearly evident why particular candidates are excluded or included in the list of novel miRNAs as well as the associated FPR estimates.

It is unclear to what extent the other prediction tools actively exclude Rfam-annotated RNA species. Here, miRdentify has no prior knowledge of genome annotation, and miRdentify seemingly demarcates miRNA from other small RNA species, e.g. snoRNA and tRNA, with high efficiency. In contrast, miRDeep2 predicts several non-miRNA Rfam species as miRNAs. One possible explanation is that read count has a high impact on the miRNA score produced by miRDeep2 and miRDeep_star, having a 0.93 and 0.99 Pearson correlation between score and read count, respectively (Supplementary Table S2). Thus, this approach seems to be very exposed to annotation of highly abundant non-miRNA species.

Regarding flexibility, the pre-defined criteria used in miRdentify, such as a pre-miRNA size between 46 and 80 nucleotides, are customizable values, and this enables miRdentify to also predict longer miRNA species, which is beneficial for the typically much longer plant pre-miRNAs. To our knowledge, this is not a possible feature in the other available prediction algorithms.

Finally, unlike miRdeep_star and miRanalyzer, which are only available as pre-compiled java applications, miRdentify is available both as open source and as a pre-compiled user-friendly windows-based application. This enables other researchers and programmers to implement, adapt and improve miRdentify to their specific needs.

In conclusion, as existing tools seem to focus on the quantity of miRNAs predicted and the associated number of reads rather than the quality of the prediction, we believe that high-stringency tools like miRdentify are of particular interest for the miRNA community especially when sequencing depth is constantly increasing.

## SUPPLEMENTARY DATA

Supplementary Data are available at NAR Online.

SUPPLEMENTARY DATA
