# Supplementary material for: miRdentify: high stringency miRNA predictor identifies several novel animal miRNAs
Source: Nucleic Acids Res. 2014 Jul 22;42(16):e124. doi: 10.1093/nar/gku598 (PMC4176371; doi:10.1093/nar/gku598)
Supplement: SUPPLEMENTARY DATA [file supp_42_16_e124__index.html]

miRdentify: high stringency miRNA predictor identifies several novel animal miRNAs — SUPPLEMENTARY DATA 

# miRdentify: high stringency miRNA predictor identifies several novel animal miRNAs

## SUPPLEMENTARY DATA

**Files in this Data Supplement:**

- SUPPLEMENTARY DATA
- Supplementary Table 2
- Supplementary Table 1
- Supplementary Table 3
- Supplementary Table 4
