# Supplementary material for: miRdentify: high stringency miRNA predictor identifies several novel animal miRNAs
Source: Nucleic Acids Res. 2014 Jul 22;42(16):e124. doi: 10.1093/nar/gku598 (PMC4176371; doi:10.1093/nar/gku598)
Supplement: SUPPLEMENTARY DATA [file supp_gku598_SI_revised.pdf]

Supplementary Information

Supplementary Fig 1-13 (enclosed below)

Supplementary Table 1: Xlsx-file; Overview of datasets used in predictions

Supplementary Table 2: Xlsx-file; Output from prediction methods

Supplementary Table 3: Xlsx-file; Output from miRdentify prediction in other organisms

Supplementary Table 4: Xlsx-file; Shell commands used

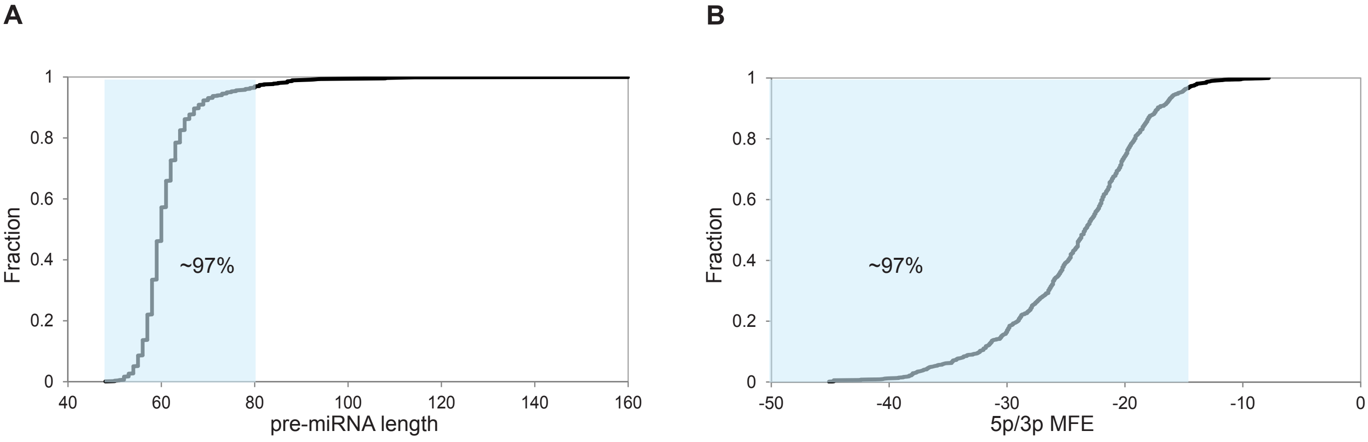

Supplementary Figure 1: miRBase annotated miRNAs. A+B) Cumulative plot of annotated pre-miRNA length **(A)** and the minimal free energy (MFE) of 5p-3p arm duplexes **(B)**. Pre-miRNAs were retrieved from miRBase version 20, and only pre-miRNAs with 5p and 3p mature annotation were considered to confidently assess both the pre-miRNA length and duplex MFE, n=909. As highlighted, the vast majority of pre-miRNA species is 46-80 nucleotides in length and exhibit a duplex MFE below -14 kcal/mol.

A

$$\sum \frac{D_i \times R_i}{R_t},$$ where R_i is sum of reads with 5' end at position D_i, *and* R_t is total sum of reads within window

B

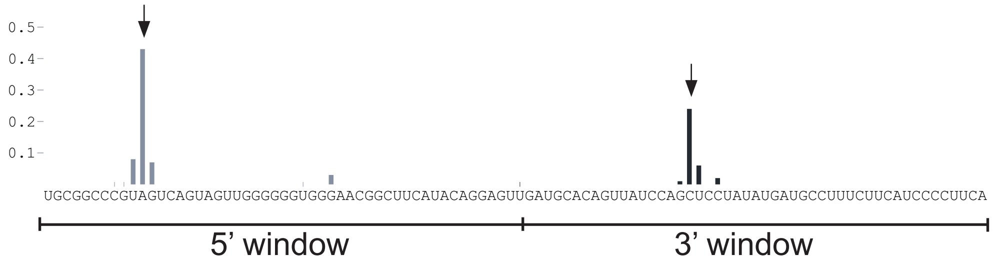

Supplementary Figure 2: Heterogeneity. A) All reads mapped onto the pre-miRNA sequence (including 10 nt up- and downstream) are divided into 5p and 3p window reads based on their midpoint position. Then for all 5p and 3p read, respectively, the 5' distance (D_i) in nucleotides to the most predominant 5p/3p read (the assigned guide/passenger strand) is multiplied by the read count (R_i). These values are then summed and divided by total reads within window (R_t). **B)** Frequency plot of 5'ends assigned as 5p-arm reads (light grey) or 3p-arm reads (dark grey) in the miR-337 locus. Guide and passenger strand 5' ends are marked by arrows.

A

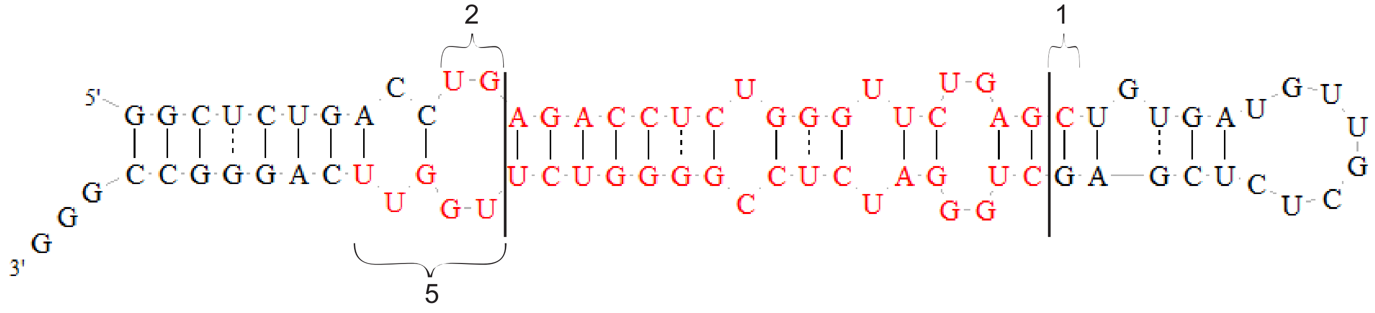

3p overhang = +5-2 = +3

5p overhang = +1-0 = +1

Supplementary Figure 3: Overhangs. Based on the most predominant 5p and 3p arm reads (guide and passenger strands), the overhang is assessed by their duplex-formation within the pre-miRNA context. The number of nucleotides flanking the first and last base-pair between guide and passenger-strands (shown by Curly brackets) are counted and subtracted. Positive score indicates 3’ overhang whereas negative score indicates 5’ overhang.

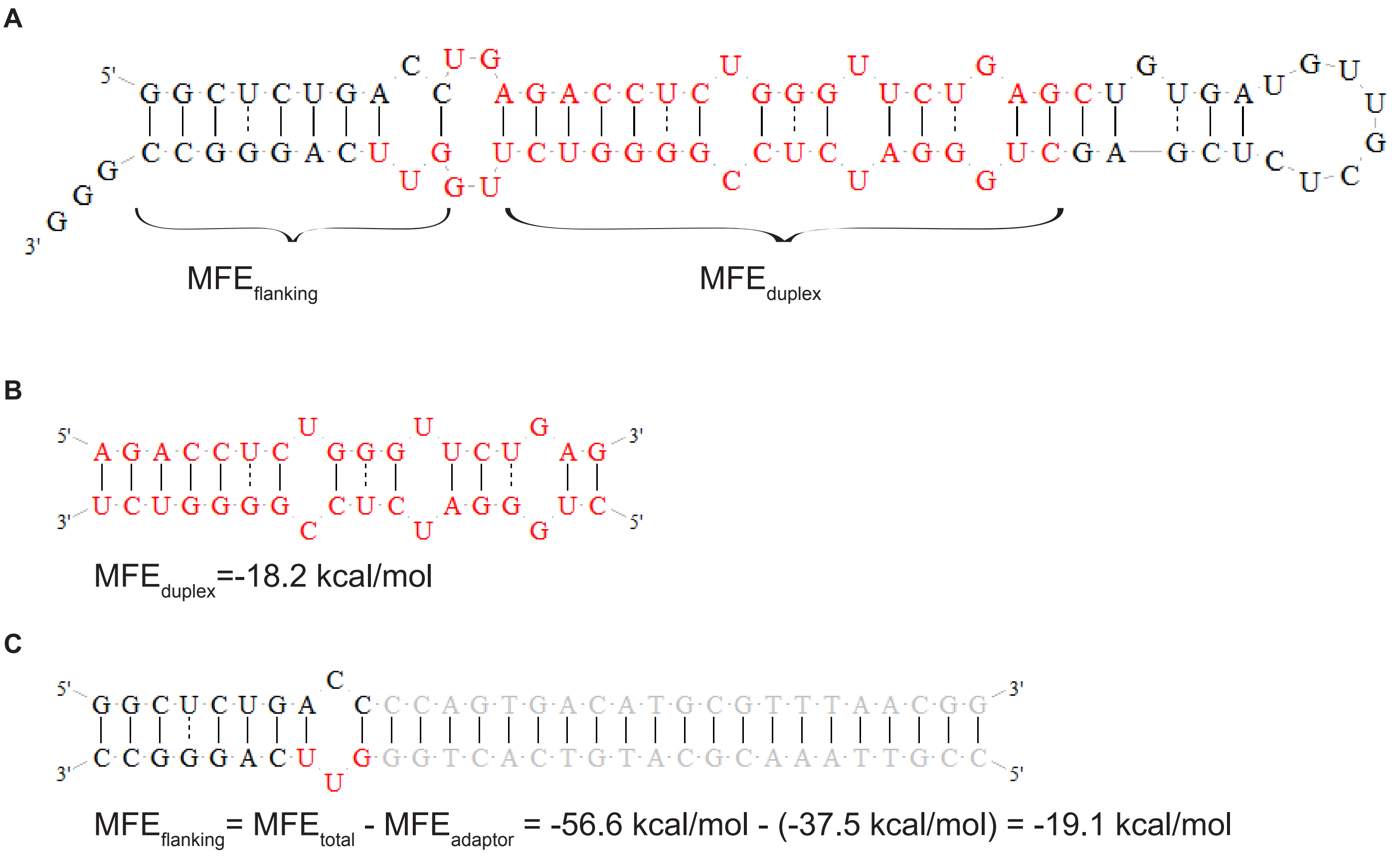

Supplementary Figure 4: Thermodynamic. A) Based on the most predominant 5p and 3p arm reads (guide and passenger strands, in red), the thermodynamic properties of the miRNA are assessed in context of the pre-miRNA structure using the MultiRNAfold version 1.1 algorithm. **B)** In case of duplex minimal free energy (MFE), only the nucleotides involved in duplex-formation with the opposed mature strand are used subjected to MFE evaluation. In the depicted example, the free energy of the duplex is -18.2 kcal/mol. **C)** In case of flanking MFE, the calculation is based on 10 nt flanking sequence on each side defined respectively by the 5'end of the 5p and by the 3'end of the 3p arm excluding the overhang. The flanking sequences are attached to an adaptor sequence (in grey) to align the sequences as in the pre-miRNA structure. The resulting MFE is the difference between the adaptor sequence alone ($MFE_{adaptor}$), and the chimeric sequence (MFE_{total}).

A

```
CAGCCGUGGCCAUCUUACUGGGCAGCAUUGGAUGGAGUCAGGUCUCUAAUACUGCCUGGUAAUGAUGACGGCGGAGCCC
...((((.(.((((((((..(((((.((((((.((.......)))))))).)))))..)))).)))).).)))).....
```

B

$-\sum F_N \times \log_2(F_N)$, where N is ‘AA’, ‘AT’, ‘AG’,..., and F_N is the frequency of N in pre-miRNA sequence.

C

$-\sum F_S \times \log_2(F_S)$, where S is ‘(...’, ‘.(...’, ‘..(.’,..., and F_S is the frequency of S in pre-miRNA structure.

Supplementary Figure 5: Entropy. A) The primary sequence and dot-bracket structure representation of a pre-miRNA including 10 nt flanking sequence on each side. **B)** Nucleotide entropy is based on the frequency of all dinucleotides (F_N) and calculated as shown. **C)** Structural entropy is based on the frequency of structural 4mers (F_s) and calculated as shown.

A

```
hsa-mir-200b MI0000342       CAGCCGUGGCCAUCUUACUGGGCAGCAUUGGAUGGAGUCAGGUCUCUAAUACUGCCUGGUAAUGAUGACGGCGGAGCCC
                             ...((((.(.((((((((..(((((.((((((.((.......)))))))).)))))..)))).)))).).)))).....
                             ..............................................UAAUACUGCCUGGUAAUGAUGACa......... 204615
                             ..............................................UAAUACUGCCUGGUAAUGAUGACu......... 151388
                             ...............................................AAUACUGCCUGGUAAUGAUGACu......... 41196
                             ..............................................UAAUACUGCCUGGUAAUGAUGAa.......... 28161
                             ..............................................UAAUACUGCCgGGUAAUGAUGA........... 27634
                             ..............................................UAAUACUGCCUGGUAAUGAUGu........... 20601
                             ..............................................UAAUACUGCCUGGUAAUGAUa............ 17836
                             ...............................................AAUACUGCCUGGUAAUGAUGACa......... 15283
                             ..............................................UAAUACUGCCUGGUAAUGAUGAu.......... 9373
                             ..............................................UAAUACUGCCUGGcAAUGAUGAC.......... 7875
                             ..............................................UAAUgCUGCCUGGUAAUGAUGAC.......... 7778
                             ..............................................UAAUACUGCCUGGgAAUGAUGAC.......... 7057
                             ..............................................UAAUACUGCCUGGaAAUGAUGAC.......... 6934
                             ..............................................UAAUACUGCCUGGcAAUGAUGA........... 6679
                             ................................................AUACUGCCUGGUAAUGAUGACu......... 6342
                             ..............................................UAAUACUGCCUGGgAAUGAUGA........... 6280
                             ..............................................UAAUgCUGCCUGGUAAUGAUGA........... 5911
                             ..............................................UAAUACUGCCUGGaAAUGAUGA........... 5610
                             ..............................................UAAUACUuCCUGGUAAUGAUGAC.......... 5483
                             ..............................................UAAUACUGCCUGGUAAUGAa............. 4693
                             ..............................................UAAUACUuCCUGGUAAUGAUGA........... 4239
                             ..............................................UAAUACUGCCUGGUAAUGAUGACc......... 4203
                             ..............................................UAAUACUGCCUGGcAAUGAUG............ 3333
                             ..............................................UAAUACUGCCUGGUAAUGAUu............ 3233
                             ..............................................UAAUACUGCCUGGgAAUGAUG............ 2939
                             ..............................................UAAUACUGCCUGGUAAUGAUaA........... 2894
                             ..............................................UAAUgCUGCCUGGUAAUGAUG............ 2755
                             ..............................................UAAUACUGCCUGGUAAUGcUGAC.......... 2724
                             ..............................................UAAUACUGCCUGGaAAUGAUG............ 2492
                             ..............................................UAAUACUGCCUGGUAAUGcUGA........... 2452
                             ..............................................UAAUACUGCCUGGUAAUGAUGg........... 2201
                             ..............................................UAAUACUuCCUGGUAAUGAUG............ 2017
                             ..............................................UAAUACUGCCUGGUAAUGAUGAg.......... 2012
                             ................................................AUACUGCCUGGUAAUGAUGACa......... 1948
                             ..........CAUCUUACUGGGCAGCAUUGGAa.............................................. 1826
                             ...
```

B

$$\frac{R_m}{R_t + R_m}$$, where R_m is the sum of mismatched reads (in red) and R_t is the sum of tailied reads (green).

Supplementary Figure 6: Tailing. A) Reads are mapped against the pre-miRNA sequence allowing a single nucleotide mismatch. For all mismatched reads (mismatch is denoted in lowercase bold), terminal A/U tailing (read count shown in green) is separated from mismatches elsewhere (read count shown in red). **B)** The tailing score is calculated as the ratio between A/U-tailed reads and the total count of single mismatched reads.

A

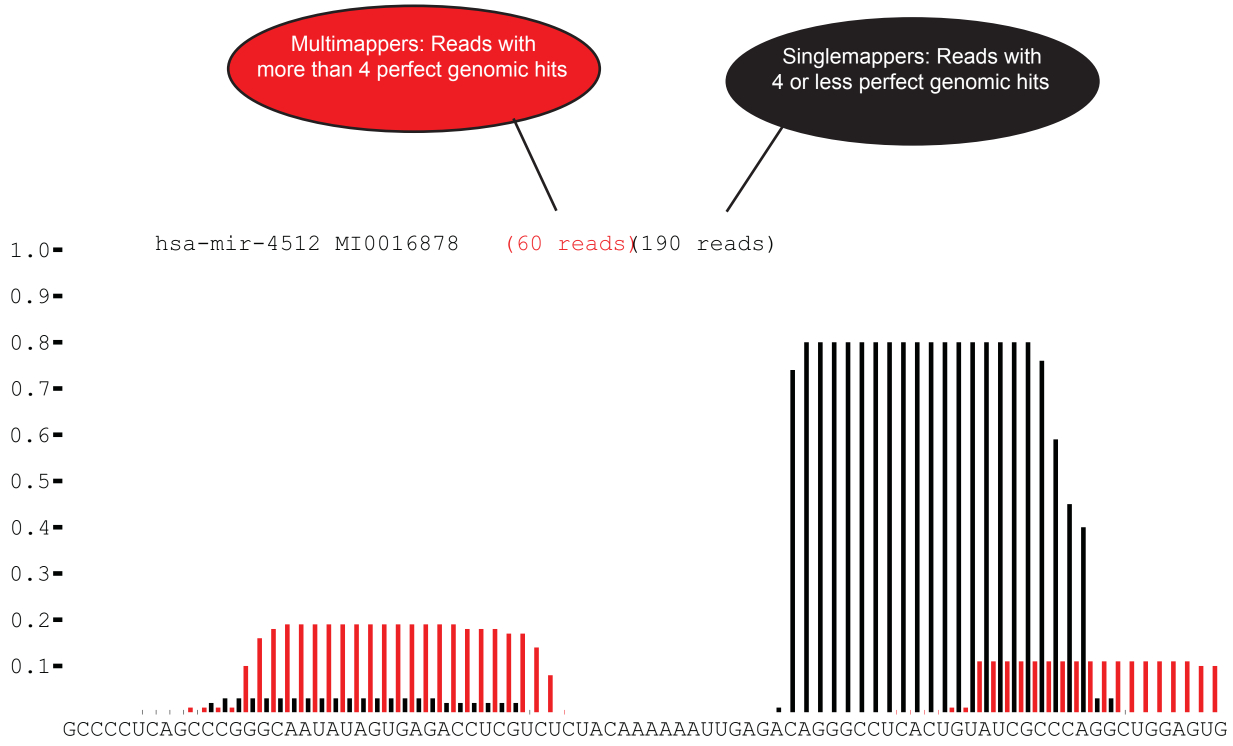

B

$\frac{R_m + R_s}{R_s}$, where R_m is the sum of multimapping reads and R_s is the sum of singlemapping reads.

Supplementary Figure 7: Multimapping. A) Read density plot depicting single mappers (black; <= 4 genomic hits) and multimappers (red; >4 genomic hits) on the miR-4512 locus. **B)** The multimapping score is calculated as the ratio between total reads (R_m+R_s) and single mappers (R_s).

A

**Relative cutoff**	**0.01**	**0.02**	**0.03**	**0.04**	**0.05**	**0.06**	**0.07**	**0.08**	**0.09**	**0.1**	**0.11**	**0.12**	**0.13**	**0.14**	**0.15**	**0.16**	**...**
5' end heterogeneity (5p arm)	6.48	5.32	4.38	3.58	2.89	2.53	2.12	1.86	**1.57**	1.42	1.34	1.22	1.12	1.04	1.00	0.95	**...**
5' end heterogeneity (3p arm)	6.25	5.05	3.77	3.02	2.54	2.22	1.88	1.73	**1.42**	1.33	1.20	1.07	1.03	1.00	0.92	0.88	**...**
Overhang (5p arm)	[-13:15]	[-7:11]	[-4:7]	[-3:6]	[-2:5]	[-2:4]	[-1:4]	[-1:4]	**[-1:3]**	[0:3]	[0:3]	[0:3]	[0:3]	[0:3]	[0:3]	[0:3]	...
Overhang (3p arm)	[-12:15]	[-6:11]	[-4:7]	[-3:6]	[-2:5]	[-1:4]	[-1:4]	[-1:4]	**[-1:3]**	[0:3]	[0:3]	[0:3]	[0:3]	[0:3]	[0:3]	[0:3]	...
MFE (duplex)	0.00	-4.54	-8.20	-10.88	-12.14	-12.64	-13.40	-13.90	**-14.50**	-14.87	-15.18	-15.58	-15.79	-16.10	-16.21	-16.51	**...**
MFE (flanking sequence)	-1.41	-2.01	-2.70	-3.33	-3.80	-4.20	-4.60	-4.70	**-4.90**	-5.10	-5.30	-5.50	-5.70	-5.90	-6.20	-6.31	**...**
Nucleotide entropy	3.18	3.30	3.37	3.42	3.46	3.47	3.50	3.52	**3.54**	3.54	3.55	3.57	3.58	3.59	3.60	3.60	**...**
Structural entropy	4.48	4.42	4.36	4.33	4.31	4.28	4.25	4.23	**4.22**	4.20	4.19	4.18	4.17	4.16	4.14	4.13	**...**
Tailing factor	0.02	0.06	0.09	0.11	0.14	0.17	0.19	0.20	**0.21**	0.24	0.25	0.27	0.29	0.30	0.31	0.32	**...**
Multimap factor	1.92	1.35	1.18	1.08	1.04	1.03	1.02	1.01	**1.01**	1.01	1.00	1.00	1.00	1.00	1.00	1.00	**...**

B

**Relative cutoff**	**0.01**	**0.02**	**0.03**	**0.04**	**0.05**	**0.06**	**0.07**	**0.08**	**0.09**	**0.1**	**0.11**	**0.12**	**0.13**	**0.14**	**0.15**	**0.16**	**...**
5' end heterogeneity (5p arm)	0.13	0.21	0.32	0.43	0.54	0.60	0.66	0.70	**0.73**	0.75	0.76	0.78	0.79	0.79	0.81	0.81	**...**
5' end heterogeneity (3p arm)	0.14	0.25	0.41	0.52	0.61	0.66	0.70	0.72	**0.76**	0.77	0.79	0.80	0.80	0.80	0.82	0.82	**...**
MFE (duplex)	0.00	0.34	0.52	0.64	0.69	0.71	0.74	0.76	**0.78**	0.80	0.81	0.82	0.83	0.83	0.84	0.85	**...**
MFE (flanking sequence)	0.05	0.11	0.15	0.20	0.23	0.26	0.30	0.31	**0.32**	0.34	0.36	0.38	0.39	0.41	0.43	0.45	**...**
Nucleotide entropy	0.04	0.07	0.10	0.12	0.15	0.16	0.18	0.20	**0.22**	0.23	0.24	0.26	0.27	0.29	0.30	0.31	**...**
Structural entropy	0.06	0.10	0.15	0.18	0.20	0.23	0.27	0.29	**0.31**	0.33	0.34	0.36	0.37	0.39	0.41	0.42	**...**
Tailing factor	0.40	0.43	0.46	0.49	0.52	0.56	0.58	0.58	**0.61**	0.63	0.66	0.67	0.67	0.69	0.69	0.70	**...**
Multimap factor	0.04	0.06	0.10	0.17	0.26	0.32	0.37	0.40	**0.42**	0.43	0.44	0.45	0.45	0.45	0.46	0.46	**...**
Overhangs	0.19	0.42	0.56	0.63	0.70	0.76	0.78	0.78	**0.82**	0.86	0.86	0.86	0.86	0.86	0.86	0.86	**...**
																	**...**
Total Candidates	31117	31117	31117	31117	31117	31117	31117	31117	**31117**	31117	31117	31117	31117	31117	31117	31117	**...**
Expected positive	9422.9	2855.4	854.9	283.6	99.2	43.0	20.6	13.9	**6.7**	3.7	2.6	1.9	1.6	1.3	1.0	0.8	**...**
Observed positive	9861	4824	2942	2123	1686	1405	1218	1114	**933**	791	719	639	589	548	492	461	**...**
FPR	0.956	0.592	0.291	0.134	0.059	0.031	0.017	0.012	**0.007**	0.005	0.004	0.003	0.003	0.002	0.002	0.002	**...**

C

Expected Positive = Π(1-Parameter Strength) x Total Candidates

= (1-0.13)x(1-0.14)x(1-0.00)x(1-0.05)x(1-0.04)x(1-0.06)x(1-0.40)x(1-0.04)x(1-0.19) x 31117

≈ 9423

FPR = Expected Positive / Observed Positive

= 9423 / 9861

= 0.956

Supplementary Figure 8: Parameter strengths and false positive rates. A) Table with absolute cutoff values for each relative cutoff fraction. **B)** Table with the parameter strengths for each relative cutoff fraction. The expected number of positive candidates is shown below (Π(1-strengths) x total number of candidates) along with the actual observed number of positive candidates. The false positive rates are calculated as the ratio between expected and observed positive candidates. The 0.09 cutoff column (FPR <= 0.01) is marked in bold. **C)** Example of FPR calculation using the 0.01 cutoff.

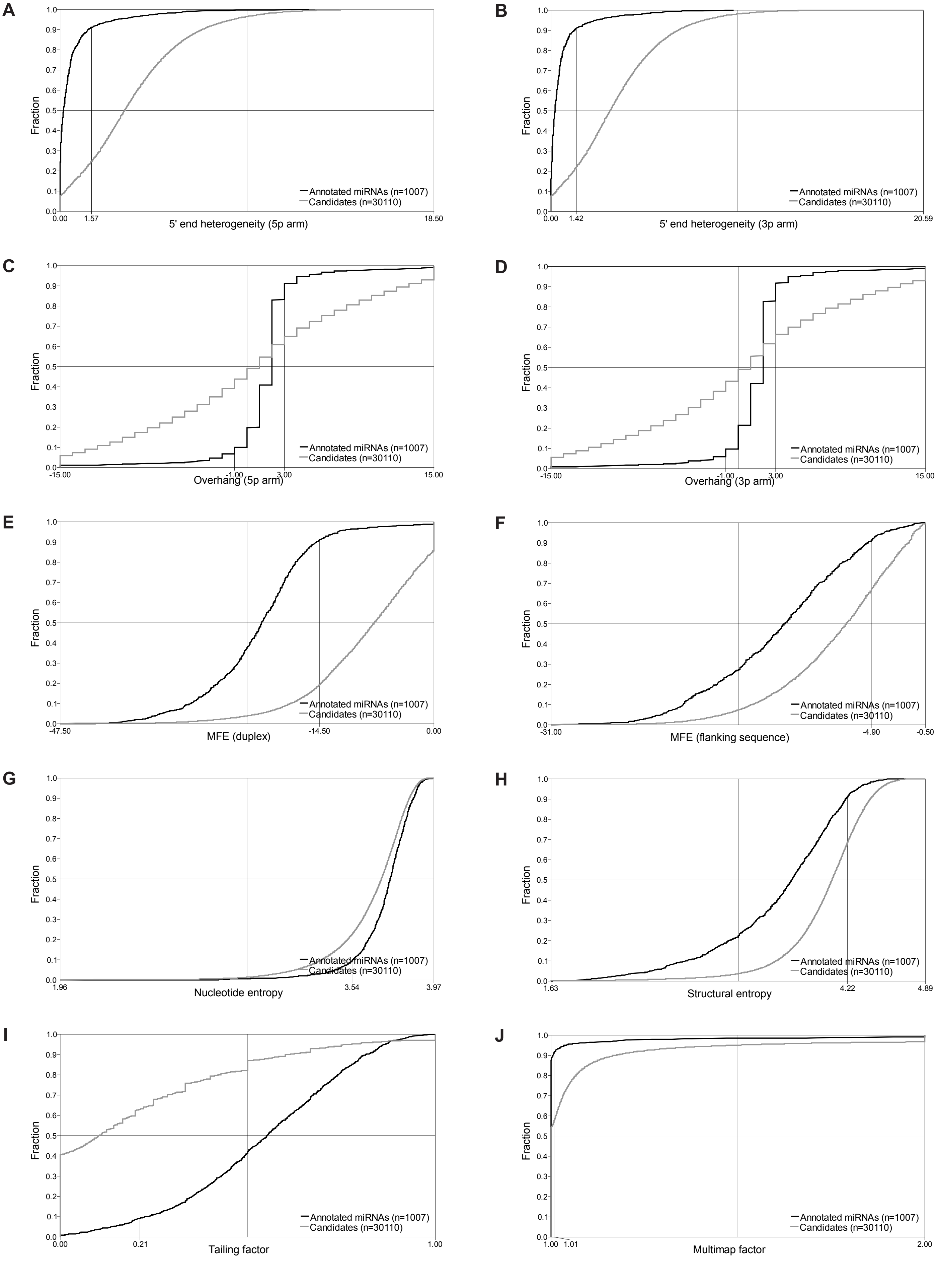

Supplementary Figure 9: Parameters with cutoff. A) As in Fig 2 including the 0.09 relative cutoff fraction value for each parameter.

A

Dataset 2	#Predicted	Hairpin length (nts)	Rfam	Repeatmask	miR-Abela	miRFinder	miRPara	miREval	MiPred	CID-miRNA	AVERAGE
**miRBase***	823	60.1 ± 4.9	0.6%	12.6%	63.2%	89.8%	91.3%	85.4%	82.9%	82.3%	82.5%
**miRdentify**	94	59.9 ± 4.1	0.0%	20.2%	53.2%	89.4%	93.6%	89.4%	89.4%	85.1%	83.3%
**miRDeep2 (score ≥ 10)**	362	61.1 ± 7.9	14.4%	27.3%	35.6%	74.0%	77.3%	87.6%	65.5%	56.1%	66.0%
**miRDeep_star (score ≥ 10)**	487	58.8 ± 4.5	1.8%	18.8%	33.3%	68.2%	79.7%	87.9%	59.8%	56.7%	64.2%
**miRanalyzer**	880	62.3 ± 5.7	1.0%	26.0%	54.7%	91.4%	86.1%	85.3%	77.0%	78.0%	78.8%
**miReap**	1783	59.3 ± 6.0	0.4%	32.0%	41.2%	77.9%	86.1%	75.0%	67.3%	60.3%	68.0%

* The miRBase-annotated miRNAs detected by miRdentify

B

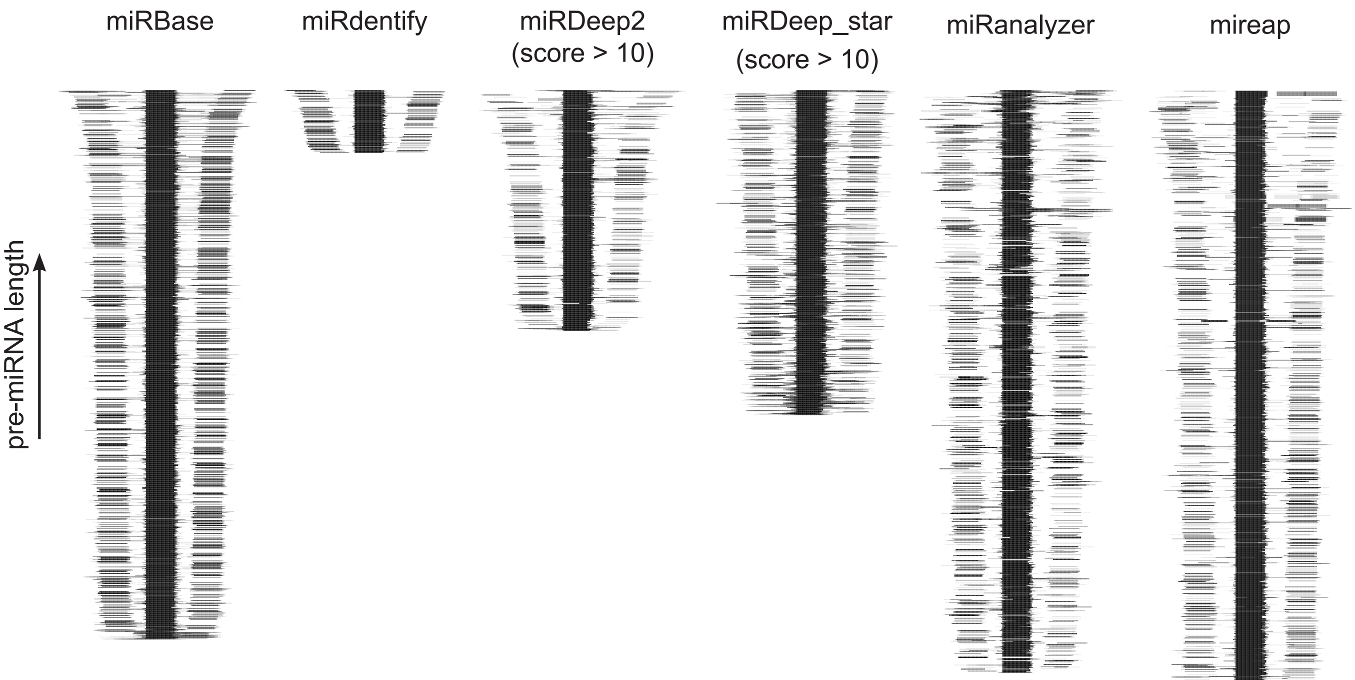

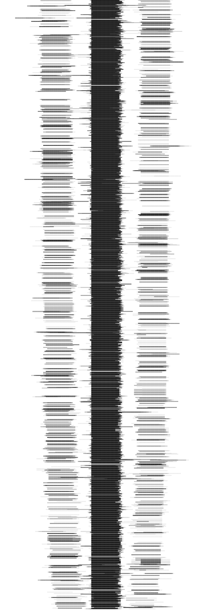

C

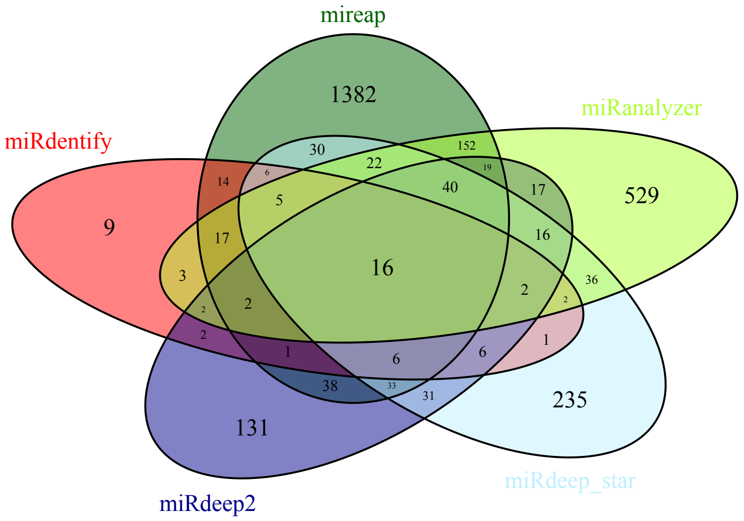

D

Duration of prediction	Hours
Dataset 1	
**miRdentify**	4
**miRDeep2**	12
Dataset 2	
**miRdentify**	<1
**miRDeep2**	7
**miRDeep_star**	19
**miRanalyzer**	24
**miReap**	<1*

*** Excluding mapping the reads**

Supplementary Figure 10: Comparing prediction with other algorithms using dataset 2. A) Comparison on methods as in Fig 5A. **B)** Distribution of reads on pre-miRNA as in Fig 5B. **C)** Venn diagram showing the overlap between predictions. **D)** Duration of predictions.

A

Dataset 1	#Predicted	Hairpin length (nts)	Rfam	Repeatmask	miR-Abela	miRFinder	miRPara	miREval	MiPred	CID-miRNA	AVERAGE
**miRBase (FDR < 0.01)**	503	60.2 +- 4.1	0.6%	13.7%	73.6%	94.6%	94.0%	89.5%	93.4%	90.5%	89.3%
**miRBase (FDR > 0.01)**	504	60.1 +- 5.7	1.0%	12.1%	48.8%	83.9%	87.9%	78.2%	68.7%	69.2%	72.8%

B

Dataset 1	#Predicted	Hairpin length (nts)	Rfam	Repeatmask	miR-Abela	miRFinder	miRPara	miREval	MiPred	CID-miRNA	AVERAGE
**miRdentify**	420	60.4 ± 5.4	0.2%	21.2%	53.3%	86.9%	93.6%	89.5%	81.9%	74.3%	79.9%
**miRdentify (Merge Heterogeneity)**	288	60.4 ± 5.4	0.3%	23.6%	57.6%	88.5%	95.5%	86.8%	84.0%	76.7%	81.5%

Supplementary Figure 11: Comparing prediction subsets. A) Comparing the excluded (FPR > 0.01) and the included (FPR < 0.01) annotated miRNAs, as in Fig 5A. **B)** Comparing prediction with or without merging the 5' end heterogeneity scores in FPR assessment.

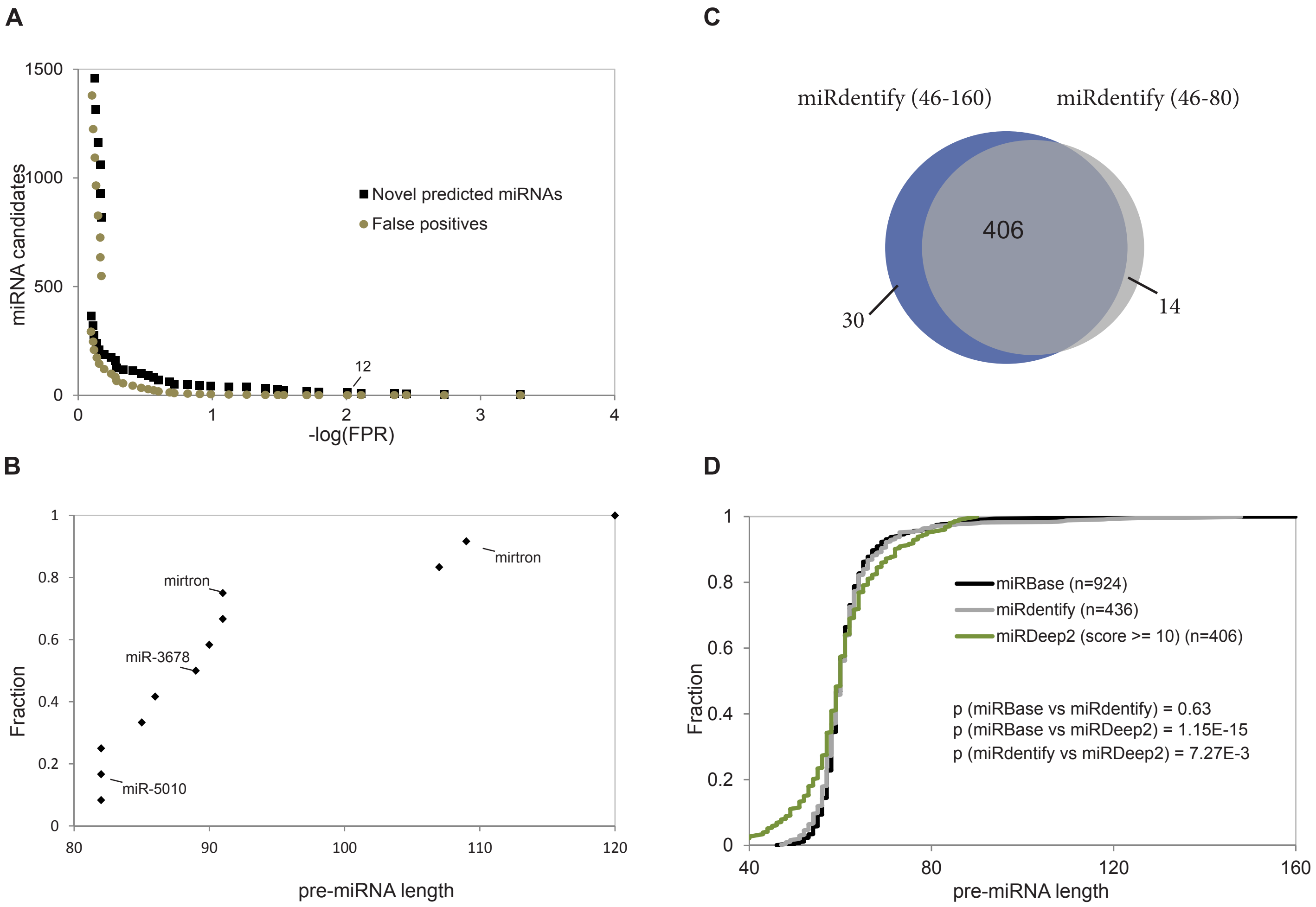

Supplementary Figure 12: Prediction of miRNAs from long pre-miRNA hairpins. A) Scatterplot with observed positive candidates and expected false positives as a function of false positive rate (FPR). 16 candidates pass the FPR<= 0.01 criterion. **B)** Cumulative plot of the predicted long pre-miRNAs. **C)** Venn-diagram depicting the overlap between miRdentify predictions allowing a pre-miRNA size-range of 46-80 nucleotides and a size-range of 46-160 nucleotides. **D)** Cumulative plot of size distribution of miRBase miRNA with 5p and 3p arm annotation, miRdentify predicted miRNAs in 46-160 nucleotide size-range and miRdeep2 predicted miRNAs. P-values are based on two-sided Kolmogorov-Smirnov tests.

A

Parameters	Hsa	Mmu	Dme	Cel
5' end heterogeneity (5p arm)	1.57	1.14	1.43	1.24
5' end heterogeneity (3p arm)	1.42	0.98	0.96	1.46
Overhang (5p arm)	[-1;3]	[-1;3]	[0;3]	[0;3]
Overhang (3p arm)	[-1;3]	[-1;3]	[0;3]	[0;3]
MFE (duplex)	-14.50	-13.83	-11.88	-13.05
MFE (flanking sequence)	-4.90	-3.53	-2.47	-1.55
Nucleotide entropy	3.54	3.58	3.61	3.62
Structural entropy	4.22	4.28	4.28	4.29
Tailing factor	0.21	0.22	0.02	0.03
Multimap factor	1.01	1.00	1.00	1.16

B

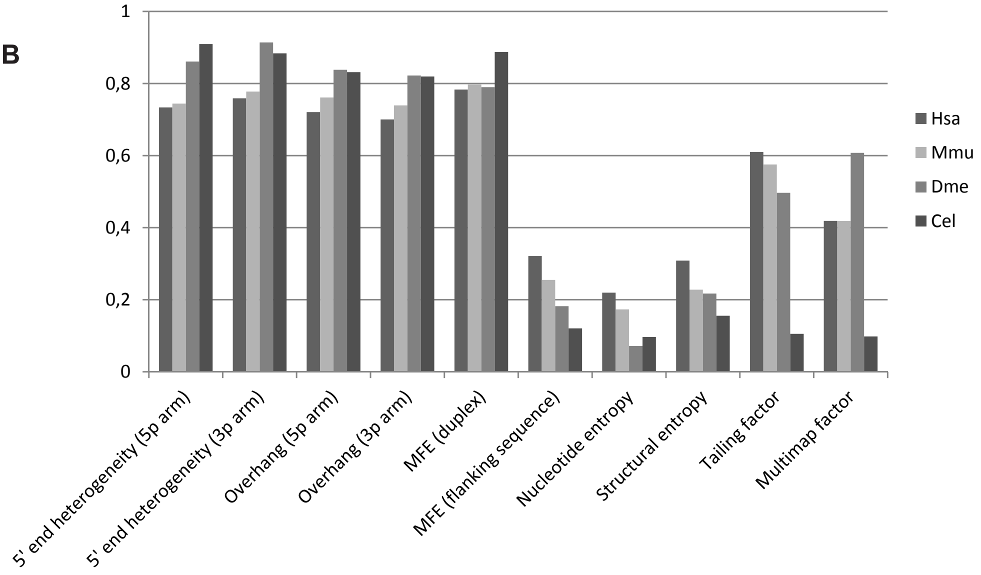

C

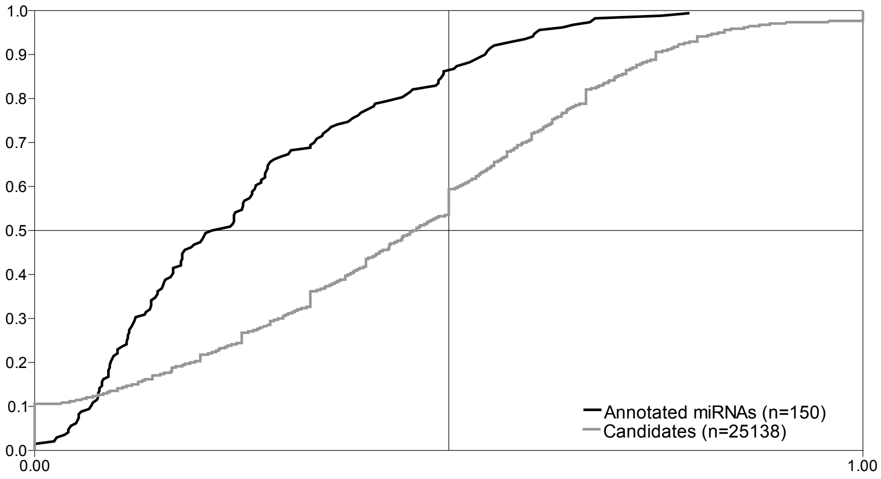

Supplementary Figure 13: Compare parameter strength across species. A) Table of parameter cutoff scores obtained for human (hsa), mouse (mmu), fruit fly (dme) and nematodes (cel). **B)** Histogram depicting the parameter strength across the four species. **C)** Cumulative fraction plot of tailing scores obtained from *C. elegans.*
